# Vaccination inequities among children 12–23 months in India: An analysis of inter-state differences

**DOI:** 10.1016/j.jvacx.2023.100310

**Published:** 2023-05-05

**Authors:** Octavia K. Goodman, Abram L. Wagner, Dakota Riopelle, Joseph L. Mathew, Matthew L. Boulton

**Affiliations:** a College of Health Sciences, Old Dominion University, 5115 Terminal Blvd, Norfolk, VA 23529, USA; b Department of Epidemiology, University of Michigan, 1415 Washington Heights, Ann Arbor, MI 48109, USA; c Department of Epidemiology, University of Michigan, 1415 Washington Heights, Ann Arbor, MI 48109, USA; d PGIMER, QQ7G+JC4, P.G.I, Sector 12, Chandigarh 160012, India; e Department of Epidemiology, School of Public Health, University of Michigan, 1415 Washington Heights, Ann Arbor, MI 48109, USA; f Department of Internal Medicine, Infectious Diseases Division, Michigan Medicine, 1500 East Medical Center Drive, Ann Arbor, MI 48109, USA

**Keywords:** India, Vaccination, Risk factors, Disparities, Infants

## Abstract

**Background:**

Previous research has shown that socioeconomic and demographic risk factors in children are additive and lead to increasingly negative impacts on vaccination coverage. The goal of this study is to examine if different combinations of four risk factors (infant sex, birth order, maternal education level, and family wealth status) vary by state among children 12–23 months in India and to determine the impact of ≥ 1 risk factor on differences in state vaccination rates.

**Methods:**

Using data from the National Family Health Survey (NFHS) conducted in India between 2005 and 2006 (NFHS-3) and 2015–2016 (NFHS-4), full vaccination of children 12–23 months was examined. Full vaccination was defined as receipt of one dose of bacillus Calmette–Guérin (BCG), three doses of diphtheria-pertussis-tetanus vaccine (DPT) vaccine, three doses of oral polio vaccine (OPV), and one dose of measles-containing vaccine (MCV). Associations between full vaccination and the four risk factors were assessed using logistic regression. Data were analyzed by the state of residence.

**Results:**

A total of 60.9% of children 12–23 months were fully vaccinated, in NFHS-4, ranging from 33.9% in Arunachal Pradesh to 91.3% in Punjab. In NFHS-4, the odds of full vaccination across all states were 15% lower among infants with 2 risk factors versus 0 or 1 risk factors (OR: 0.85, 95% CI: 0.80–0.91), and 28% lower among infants with 3 or 4 risk factors versus 0 or 1 risk factor (OR: 0.72, 95% CI: 0.67–0.78). Overall, the absolute difference in the full vaccination coverage in those with > 2 vs < 2 risk factors decreased from −13% in NFHS-3 to −5.6% in NFHS-4, with substantial variation across states.

**Conclusions:**

Disparities in full vaccination exist among children 12–23 months experiencing > 1 risk factor. Indian states that are more populous or located in the north were more likely to have greater disparities.

## Highlights

• India’s full childhood vaccination was 44.7% in 2005–2006 and 60.9% in 2015–2016.
• Disparities exist based on sex, birth order, maternal education, and wealth status.
• More populous and northern Indian states showed greater vaccination disparities.
• There is a need to reduce inequities in childhood vaccination rates.
• Future research should examine impact of combinations of risk factors on vaccination.

## Introduction

The World Health Organization (WHO) launched the Expanded Programme on Immunization (EPI) in 1974 to ensure that infants across the world had access to the four recommended vaccines that protect against serious childhood illnesses. These included the bacillus Calmette-Guerin (BCG), diphtheria-pertussis-tetanus vaccine (DPT) vaccine, polio vaccine, and measles vaccine [1]. India adopted the WHO EPI in 1978, however the full complement of six antigens recommended by the WHO was not introduced until 1985 [2], [3]. Although childhood immunization is historically considered one of the most successful public health interventions, many low- and middle-income countries (LMICs) struggle to achieve vaccination targets. In fact, global vaccination coverage of the recommended childhood vaccines has declined from 86% in 2019 to 83% in 2020 [4]. It is estimated that 23 million children under one year of age did not receive basic vaccines in 2020, which represents an increase of 3.7 million infants compared to the previous year and the highest number seen since 2009 [4]. Moreover, roughly two-thirds of children under one year old who did not receive any vaccines live in just 10 countries, including India [1]. In 2019, India had the second greatest number of completely unvaccinated children, accounting for over 1 million zero-dose infants [1].

In examining equity in vaccination programs, previous research in India has explored the impact of individual level risk factors on vaccination rates among children [5], [6], [7]. Many of these studies have used the National Family Health Survey (NFHS) or District Level Household Survey (DLHS) data. These have shown lower vaccination coverage among infants who were female [8], higher birth order [8], low or no maternal education [9], [10], and families with lower socioeconomic status [10], [11], [12], [13]. Moreover, a review of global literature on vaccination found that several risk factors--infant sex, infant birth order, family wealth, and maternal education--strongly correlated with a child’s vaccination status [25]. In a 2020 study by Bettampadi et al. [5], the authors found that these four risk factors had an additive negative impact on infant vaccination. However, it remains unclear if these socioeconomic disparities persist across India’s state borders, especially as the national government has prioritized vaccination programs in specific underserved areas.

Programmatic and operational factors contributing to disparities in vaccination rates within India comprise differences in funding, demographics, community health programs and workers, vaccine roll outs, and linguistic and cultural diversity across states. Recognizing that a significant proportion of Indian infants remained unvaccinated or only partially vaccinated, the Government intensified efforts through the Mission Indradhanush, and subsequently Intensified Mission Indradhanush programmes. However, in a study examining the effectiveness of the Universal Immunization Programme (UIP), the launch of Mission Indradhanush in 2014, and of the Intensified Mission Indradhanush in 2017**,** the authors concluded that the immunization rates across approximately 15 Indian state and union territories were still below the national average [2]**.** Bhadoria and colleagues [2] reported that Puducherry was the only state that achieved the goal set by Mission Indradhanush of obtaining ≥ 90% immunization coverage by 2020**.** In addition to the start of the aforementioned programs, India also launched Janani Suraksha Yojana (JSY) in 2005, a conditional cash transfer program, in 2005 to encourage the use of reproductive and child health services [14]. Carvalho and colleagues [14] found that financial assistance provided by the JSY program contributed to an increase of 3.1 percentage points for a single dose of the polio vaccine and 9.1 percentage points among the proportion of fully vaccinated children, indicating an increase in immunization coverage among children.

Since the NFHS datasets have collected data across time and states, they can be used to examine changes in vaccination both over time and across states. Given that past research has identified several risk factors as important contributors to vaccination coverage on a national scale in India [5], it is important to identify if specific combinations of these risk factors persist in exacerbating inequities in vaccination at a state-level, particularly in those states which have benefited from government programs to increase vaccination coverage. The goal of this study is to examine if varying combinations of four different risk factors (infant sex, birth order, maternal education level, and family wealth status) differ by state among children 12–23 months in India and to determine their impact on differences in state vaccination rates among these children.

## Methods

We used data from the National Family Health Survey (NFHS)**,** which is a nation-wide survey conducted in households throughout India [15] as part of the Demographic and Health Surveys (DHS) program [16]. Since 1984, the DHS program has conducted over 400 surveys in 90 different countries, and provides comprehensive, primary data on maternal and infant health [16], including questions on childhood vaccination [17].

Data from the third wave (NFHS-3, collected Dec 2005 – May 2006) and fourth wave (NFHS-4, collected Jan 2015– Dec 2016**)** were used in this analysis. There were 10,074 children aged 12–23 months in NFHS-3 [18] and 51,574 in NFHS-4 [19]. Among these, the proportions alive at the time of survey were 95.1% (n = 9,582) and 95.6% (n = 49,284), respectively [18], [19]. For the purpose of this study, 9,582 children aged 12–23 months in NFHS-3 and 48,628 children aged 12–23 months in NFHS-4 were included in this analysis. The NFHS-3 only included children living in 29 states across India [18]; however, the NFHS-4 included children in 29 Indian states and seven union territories [19]. States and territories with low number of infants with zero risk factors (out of the four) in either the NFHS-3 or NFHS-4 were excluded from the analysis**.**

Infant full vaccination status was defined as the receipt of 1 dose of BCG vaccine; 3 doses of DPT; 3 doses of OPV; and 1 dose of MCV. For each vaccination, receipt was determined by the immunization date on the vaccination card, or the vaccination marked as having been received on the card. Vaccinations reported only by the mother or a response of **“**don’t know” were considered missing data. In NFHS-3, a vaccination card was available for 37.5% of infants, and in NFHS-4 for 63.2% of infants.

Infant sex, birth order, family wealth status, and maternal education were included as predictors of infant full vaccination status with female sex, low birth order, no formal maternal education, and low family wealth status serving as the referents based on prior research [5]. The negative cumulative impact of infant sex, birth order, family wealth status, and maternal education were examined by creating three groups: those with < 2 risk factors, with 2 risk factors, or with > 2 risk factors.

Data were examined for each of the states and union territories of India, although those with relatively low number of infants with zero risk factors out of the four risk factors of interest in either NFHS-3 or NFHS-4 or in both surveys were excluded from the analysis for the sake of comparability.

### Statistical analysis

We graphically display net state domestic product per capita (using 2016 data from the Reserve Bank of India) and full vaccination coverage using Datawrapper (Datawrapper, Berlin, Germany).

The relationship between number of risk factors and full vaccination was assessed using logistic regression models, with the model outputting odds ratios (ORs) and 95% confidence intervals (CIs). These models were run nationwide and for each individual state. They were also run separately for NFHS-3 and NFHS-4. The models included risk factor count as the independent variable, with no other covariates.

Subsequently, we compared results between NFHS-3 and 4 by subtracting out the full vaccination coverage between the two study dates. We also calculate the absolute difference in full vaccination coverage between those with > 2 vs < 2 risk factors for each state, and qualitatively compare these across the two study dates.

All analyses were conducted using SAS and SAS studio (SAS Institute, Cary, NC, USA). Survey weights, clusters, and strata information were included in the analyses in order to account for the survey design in NFHS-3 and NFHS-4. P-values were considered statistically significant at p < 0.05. Missing data were excluded from analysis. IRB and ethical approval were not necessary as NFHS is a publicly available dataset and no identifiable subject data were accessed, analyzed, or presented.

## Results

**Fig. 1:**
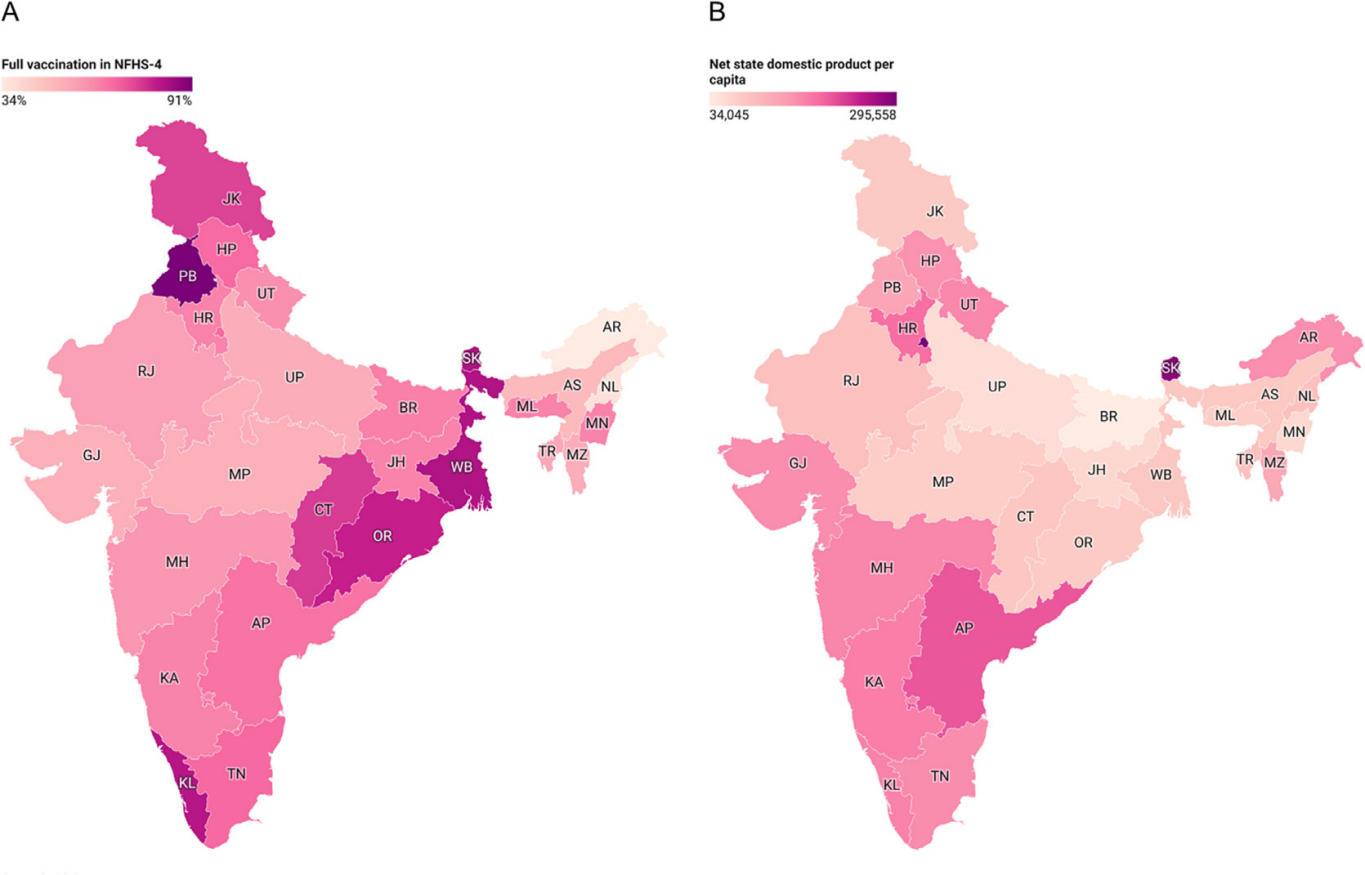
Geographic distribution of vaccination coverage by state (panel A) and net state domestic product per capita (panel B).

In the NFHS-3, the range in study sample size eligible for inclusion by state was 114 in Tripura (TR) to 1155 in Uttar Pradesh (UP) and in the NFHS-4, the range in study sample size eligible for inclusion in the study was 209 in Sikkim (SK) to 7735 in Uttar Pradesh (UP). Table 1 shows a breakdown of characteristics by children with 0, 1, 2, 3, or 4 risk factors among the 28 states that were included in the analysis. Overall, childhood full vaccination coverage was 44.7% for all Indians states, ranging from a low of 21.0% in Nagaland (NL) to a high of 82.1% in Tamil Nadu (TN) in NFHS-3. In NFHS-4, full vaccination coverage was 60.9% overall, ranging from 33.9% in Arunachal Pradesh (AR) to 91.3% in Punjab (PB). Fig. 1 shows the geographic distribution of vaccination coverage by state, with relatively low coverage in states in central India (panel A), which corresponds also to lower net state domestic product per capita (panel B).

**Table 1:** Characteristics of Indian children 12–23 months by state, NFHS-3 and NFHS-4.

		**NFHS-3 (2005**–**2006)**							**NFHS-4 (2015**–**2016)**						
**State**		**Count**	**Fully vaccinated**	**0 risk factors**	**1 risk factor**	**2 risk factors**	**3 risk factors**	**4 risk factors**	**Count**	**Fully vaccinated**	**0 risk factors**	**1 risk factor**	**2 risk factors**	**3 risk factors**	**4 risk factors**
***Overall***		*9582*	*44.7*	*1.8*	*19.5*	*44.8*	*29.9*	*4.0*	*48,628*	*60.9*	*5.2*	*26.2*	*43.0*	*23.3*	*2.3*
**Andhra Pradesh & Telangana**	AP	404	50.1	4.6	18.2	47.3	26.9	3.0	1057	66.0	3.8	25.1	39.7	28.7	2.7
**Arunachal Pradesh**	AR	152	30.1	1.2	18.9	38.5	36.6	4.8	894	33.9	4.7	23.5	41.4	26.9	3.6
**Assam**	AS	253	33.7	0.5	14.0	36.6	36.9	12.0	1935	48.7	11.0	34.6	38.0	15.9	0.5
**Bihar**	BR	381	31.4	2.0	16.8	45.7	33.2	2.3	4893	63.5	5.2	22.7	44.9	25.9	1.2
**Chhattisgarh**	CT	**NA**	**NA**	**NA**	**NA**	**NA**	**NA**	**NA**	1541	75.6	7.1	27.1	44.8	20.4	0.7
**Gujarat**	GJ	286	47.0	2.4	18.2	48.0	29.3	2.1	1409	51.9	3.3	26.6	44.9	21.7	3.6
**Haryana**	HR	210	66.1	3.3	21.7	50.5	22.6	1.8	1511	63.0	0.9	20.9	45.8	27.4	5.1
**Himachal Pradesh**	HP	180	77.6	1.4	19.3	53.5	24.4	1.4	572	68.0	3.2	26.9	44.9	24.3	0.7
**Jammu and Kashmir**	JK	230	67.4	4.7	35.5	37.0	21.0	1.8	1618	74.5	2.6	23.9	42.9	26.5	4.1
**Jharkhand**	JH	271	36.1	0.9	12.6	38.8	40.3	7.4	2394	63.0	8.9	29.1	39.6	21.5	0.9
**Karnataka**	KA	387	55.9	1.3	20.5	49.4	25.2	3.6	1508	62.7	3.7	27.7	44.1	22.3	2.3
**Kerala**	KL	194	78.7	0.0	21.7	50.5	25.3	2.6	492	82.1	0.4	24.0	50.6	25.0	0.0
**Madhya Pradesh**	MP	490	41.4	2.3	11.0	45.0	36.1	5.7	4574	51.9	6.7	28.2	42.0	21.2	1.9
**Maharashtra**	MH	583	60.6	0.2	27.3	46.0	23.0	3.4	1768	58.6	4.4	29.1	44.4	21.2	1.0
**Manipur**	MN	329	49.2	0.9	19.5	45.8	29.9	3.9	1150	62.8	6.4	31.3	44.3	17.7	0.3
**Meghalaya**	ML	209	34.4	1.7	11.8	48.7	28.7	9.1	844	62.9	5.5	30.2	40.8	22.0	1.5
**Mizoram**	MZ	152	48.1	0.0	12.5	51.4	35.5	0.6	995	52.5	2.5	21.4	48.2	27.4	0.4
**Nagaland**	NL	420	21.0	1.1	12.4	42.3	36.4	7.9	893	37.0	6.4	30.9	44.5	17.3	1.0
**Delhi**	DL	205	64.6	4.2	24.4	49.5	21.6	0.3	307	67.8	0.0	19.5	46.9	27.7	5.9
**Odisha**	OR	302	56.4	0.6	16.6	40.0	35.7	7.1	2095	79.5	8.8	33.2	39.2	17.8	1.0
**Punjab**	PB	225	65.0	4.0	33.8	45.4	16.8	0.0	1029	91.3	0.4	23.5	46.6	25.6	4.0
**Rajasthan**	RJ	328	27.6	4.2	21.7	43.8	28.0	2.3	3151	56.2	3.9	21.6	43.1	27.4	3.9
**Sikkim**	SK	124	69.2	2.0	29.5	42.6	23.1	3.0	209	82.8	1.9	28.2	44.0	22.5	2.4
**Tamil Nadu**	TN	290	82.1	2.0	26.3	44.7	22.6	4.4	1581	68.1	3.2	27.9	46.2	21.5	1.2
**Tripura**	TR	114	50.3	1.6	24.1	45.6	23.1	5.6	254	53.9	12.2	40.6	33.9	13.0	0.4
**Uttar Pradesh**	UP	1155	22.8	1.7	18.6	46.0	31.1	2.7	7735	52.5	4.7	22.9	42.2	26.3	3.9
**Uttarakhand**	UT	207	62.2	0.9	20.4	49.8	26.8	2.0	1105	60.1	2.2	24.0	46.7	23.8	3.4
**West Bengal**	WB	394	67.6	0.8	15.3	36.4	38.0	9.5	1114	83.0	10.3	34.8	37.4	16.7	0.7

Table 2, Table 3 (OR with 95% CI) represent the disparities in full vaccination coverage based on the number of risk factors in NFHS-3 and NFHS-4, respectively. In each table, a comparison of a combination of < 2, 2, or > 2 risk factors, with 2 risk factors serving as the reference group, are represented by three groups of odds ratio. In Table 2 (NFHS-3), there is a statistically significant (p < 0.0001) disparity in full vaccination by the number of risk factors among infants. In NFHS-3, the overall OR for full vaccination was 0.81 (95% CI: 0.69, 0.94) among children with 2 versus < 2 risk factors, 0.76 (95% CI = 0.67, 0.87) among infants with > 2 risk factors versus 2 risk factors, and 0.61 (95% CI = 0.52, 0.72) among infants with > 2 versus < 2 risk factors. Seven states showed statistically significant inequities based on the number of risk factors. The presence of > 2 versus < 2 risk factors was associated with a lower odds of full vaccination in Bihar (BR), Maharashtra (MH), Manipur (MN), Madhya Pradesh (MP), and Uttar Pradesh (UP). Only in Karnataka (KA) state did children with 2 risk factors have lower odds of full vaccination compared to < 2 risk factors. In contrast, three states including Bihar (BR), Meghalaya (ML), and Maharashtra (MH) showed a statistically significant association with lower odds of full vaccination among those with > 2 risk factors compared to < 2 risk factors.

**Table 2:** Odds of full vaccination by number of demographic risk factors (female sex, low birth order, no formal maternal education, and low family wealth status) by Indian state, NFHS-3 (2005–2006).

**State**	**OR for 2 vs < 2 (0 or 1) risk factors**	**OR for > 2 (3 or 4) vs 2 risk factors**	**OR for > 2 (3 or 4) vs < 2 (0 or 1) risk factors**	**P-value**
***Overall***	***0.81 (0.69, 0.94)***	***0.76 (0.67, 0.87)***	***0.61 (0.52, 0.72)***	***<0.0001***
**AP**	0.81 (0.40, 1.67)	0.72 (0.37, 1.39)	0.59 (0.30, 1.15)	0.2766
**AR**	0.51 (0.16, 1.63)	0.76 (0.32, 1.82)	0.39 (0.12, 1.24)	0.2800
**AS**	0.70 (0.30, 1.62)	0.74 (0.44, 1.26)	0.52 (0.24, 1.14)	0.1938
**BH**	0.69 (0.35, 1.37)	**0.46 (0.25, 0.83)**	**0.31 (0.14, 0.71)**	**0.0119**
**DL**	1.35 (0.61, 2.96)	1.19 (0.56, 2.52)	1.60 (0.67, 3.81)	0.5586
**GJ**	1.25 (0.68, 2.29)	0.68 (0.41, 1.14)	0.85 (0.44, 1.62)	0.3408
**HP**	0.70 (0.20, 2.45)	1.46 (0.45, 4.73)	1.02 (0.29, 3.60)	0.7784
**HR**	1.34 (0.66, 2.70)	0.74 (0.38, 1.41)	0.98 (0.44, 2.18)	0.5524
**JH**	0.61 (0.26, 1.43)	0.84 (0.46, 1.51)	0.51 (0.23, 1.17)	0.2791
**JM**	1.05 (0.57, 1.92)	0.51 (0.24, 1.09)	0.54 (0.26, 1.09)	0.1687
**KA**	**0.57 (0.35, 0.95)**	1.42 (0.89, 2.27)	0.82 (0.49, 1.37)	0.0799
**KE**	1.26 (0.56, 2.82)	0.69 (0.31, 1.54)	0.87 (0.35, 2.17)	0.6410
**MG**	0.89 (0.29, 2.73)	**0.54 (0.30, 0.99)**	0.48 (0.16, 1.49)	0.1047
**MH**	1.26 (0.75, 2.12)	**0.44 (0.26, 0.72)**	**0.55 (0.31, 0.98)**	**0.0060**
**MN**	0.55 (0.29, 1.03)	0.64 (0.39, 1.03)	**0.35 (0.17, 0.69)**	**0.0110**
**MP**	0.66 (0.38, 1.15)	0.70 (0.44, 1.11)	**0.46 (0.25, 0.83)**	**0.0355**
**MZ**	1.61 (0.49, 5.31)	0.60 (0.32, 1.14)	0.98 (0.27, 3.50)	0.2486
**NA**	0.61 (0.30, 1.23)	0.64 (0.36, 1.17)	**0.39 (0.19, 0.83)**	**0.0490**
**OR**	0.98 (0.48, 2.02)	0.76 (0.44, 1.32)	0.75 (0.37, 1.54)	0.5633
**PJ**	1.05 (0.57, 1.92)	1.51 (0.59, 3.82)	1.58 (0.63, 3.96)	0.6109
**RJ**	0.94 (0.49, 1.83)	0.86 (0.51, 1.46)	0.81 (0.42, 1.59)	0.7832
**SK**	0.69 (0.25, 1.92)	2.00 (0.53, 7.61)	1.39 (0.29, 6.72)	0.4978
**TN**	0.79 (0.32, 1.97)	0.99 (0.44, 2.20)	0.78 (0.30, 2.00)	0.8494
**TR**	0.76 (0.27, 2.11)	0.45 (0.19, 1.10)	0.34 (0.12, 1.00)	0.0980
**UC**	0.69 (0.31, 1.56)	0.73 (0.35, 1.50)	0.51 (0.20, 1.29)	0.3596
**UP**	0.72 (0.49, 1.06)	0.90 (0.65, 1.25)	**0.65 (0.43, 0.98)**	0.1138
**WB**	0.63 (0.29, 1.34)	1.14 (0.66, 1.99)	0.72 (0.32, 1.61)	0.4751

**Note:** Boldface represents statistically significant results.

**Table 3:** Odds of full vaccination by number of demographic risk factors (female sex, low birth order, no formal maternal education, and low family wealth status) by Indian state, NFHS-4 (2015–2016).

**State**	**OR for 2 vs < 2 (0 or 1) risk factors**	**OR for > 2 (3 or 4) vs 2 risk factors**	**OR for > 2 (3 or 4) vs < 2 (0 or 1) risk factors**	**P-value**
***Overall***	***0.85 (0.80, 0.91)***	***0.85 (0.79, 0.91)***	***0.72 (0.67, 0.78)***	***<0.0001***
**AP**	0.78 (0.55, 1.10)	0.95 (0.66, 1.36)	0.74 (0.51, 1.07)	0.2051
**AR**	**0.48 (0.32, 0.71)**	1.06 (0.72, 1.56)	**0.51 (0.33, 0.77)**	**0.0006**
**AS**	0.83 (0.66, 1.04)	1.06 (0.77, 1.46)	0.88 (0.65, 1.18)	0.2527
**BR**	**0.82 (0.70, 0.96)**	0.88 (0.75, 1.03)	**0.72 (0.60, 0.86)**	**0.0015**
**CT**	1.02 (0.75, 1.40)	**0.65 (0.45, 0.94)**	**0.66 (0.45, 0.98)**	**0.0522**
**GJ**	0.85 (0.62, 1.16)	0.84 (0.60, 1.16)	**0.71 (0.51, 0.98)**	0.1120
**HR**	0.78 (0.57, 1.07)	**0.72 (0.53, 0.98)**	**0.56 (0.39, 0.82)**	**0.0103**
**HP**	0.61 (0.37, 1.01)	0.99 (0.59, 1.67)	0.61 (0.35, 1.08)	0.1171
**JK**	0.99 (0.68, 1.43)	0.94 (0.68, 1.30)	0.93 (0.65, 1.32)	0.8979
**JH**	0.89 (0.71, 1.10)	0.79 (0.62, 1.02)	**0.71 (0.56, 0.89)**	**0.0157**
**KA**	1.15 (0.79, 1.68)	0.85 (0.52, 1.42)	0.98 (0.62, 1.57)	0.7274
**KL**	0.76 (0.40, 1.44)	1.30 (0.69, 2.47)	0.98 (0.48, 2.00)	0.6079
**MP**	0.97 (0.84, 1.13)	**0.79 (0.67, 0.92)**	**0.76 (0.64, 0.91)**	**0.0050**
**MH**	0.78 (0.57, 1.08)	1.19 (0.85, 1.67)	0.93 (0.65, 1.33)	0.2990
**MN**	0.85 (0.63, 1.13)	0.99 (0.69, 1.43)	0.84 (0.57, 1.23)	0.4707
**ML**	1.34 (0.97, 1.87)	0.73 (0.49, 1.09)	0.98 (0.64, 1.50)	0.1331
**MZ**	1.65 (1.00, 2.71)	1.14 (0.74, 1.76)	1.88 (1.07, 3.29)	0.0707
**NL**	1.01 (0.73, 1.40)	0.95 (0.64, 1.42)	0.96 (0.61, 1.51)	0.9734
**DL**	1.69 (0.77, 3.73)	0.69 (0.31, 1.57)	1.17 (0.42, 3.27)	0.3437
**OR**	0.68 (0.52, 0.89)	1.02 (0.72, 1.44)	**0.69 (0.49, 0.97)**	**0.0109**
**PB**	0.70 (0.34, 1.42)	0.60 (0.31, 1.17)	**0.42 (0.20, 0.86)**	0.0578
**RJ**	0.97 (0.80, 1.19)	1.22 (1.02, 1.47)	1.19 (0.96, 1.48)	0.0858
**SK**	2.36 (0.85, 6.57)	0.38 (0.14, 1.03)	0.89 (0.33, 2.42)	0.1211
**TN**	1.13 (0.87, 1.48)	0.78 (0.56, 1.07)	0.88 (0.63, 1.23)	0.2845
**TR**	1.20 (0.70, 2.06)	**0.42 (0.21, 0.86)**	0.51 (0.25, 1.03)	0.0611
**UP**	**0.78 (0.69, 0.89)**	**0.78 (0.70, 0.88)**	**0.61 (0.54, 0.70)**	**<0.0001**
**UT**	1.00 (0.72, 1.39)	0.80 (0.57, 1.11)	0.80 (0.56, 1.14)	0.3425
**WB**	0.90 (0.60, 1.36)	**0.60 (0.38, 0.95)**	**0.54 (0.35, 0.84)**	**0.0199**

**Note:** Boldface represents statistically significant results.

The NFHS-4 survey had similar national level findings as NFHS-3. Across all states for NFHS-4 (Table 3), there is a statistically significant (p < 0.0001) disparity in full vaccination by the number of risk factors among infants. The odds of full vaccination were 0.85 (95% CI: 0.80, 0.91) among children with 2 versus < 2 risk factors, 0.72 (95% CI: 0.67, 0.78) for children with > 2 versus < 2 risk factors, and 0.85 (95% CI: 0.79, 0.91) for children with > 2 versus 2 risk factors. However, 12 states showed statistically significant inequities depending on the number of risk factors. The presence of > 2 versus < 2 risk factors was associated with a lower odds of full vaccination in Arunachal Pradesh (AR), Bihar (BR), Chhattisgarh (CT), Gujarat (GJ), Haryana (HR), Jharkhand (JH), Madhya Pradesh (MP), and Uttar Pradesh (UP). Children with 2 risk factors in Arunachal Pradesh (AP), Bihar (BR), Odisha (OR), and Uttar Pradesh (UP) had lower odds of full vaccination compared to those with < 2 risk factors. Finally, children in Chhattisgarh (CT), Haryana (HR), Madhya Pradesh (MP), Tripura (TR), Uttar Pradesh (UP), and West Bengal (WB) with > 2 risk factors had a lower odds of full vaccination compared to those with 2 risk factors.

Table 4 displays the range of disparities between three risk factors and one risk factor in NFHS-3 and NFHS-4, and the difference in full vaccination coverage between the two surveys. Overall, vaccination coverage is 16.2 % higher across all Indian states and union territories in the NFHS-4 relative to the NFHS-3. A comparison of the full vaccination in the two surveys showed that in NFHS-3, vaccination coverage was −13.0% lower for those with > 2 vs < 2 risk factors, whereas this disparity was just −5.6% in NFHS-4. The states of Bihar (BH) (32.2%), Uttar Pradesh (UP) (29.7%), Rajasthan (RJ) (28.5%), Meghalaya (MG) (28.5%), Jharkhand (JH) (26.9%), Punjab (PJ) (26.3%), Odisha (aka Orissa) (OR) (23.1%), Andhra Pradesh (AP) (16.0%), West Bengal (WB) (15.4%), Assam (AS) (15.0%), Sikkim (SK) (13.6%), Manipur (MN) (13.6%), and Madhya Pradesh (MP) (10.5%) showed the highest increase in full vaccination coverage between NFHS-3 and NFHS-4. Conversely, Tamil Nadu (TN) (-14.0%), Himachal Pradesh (HP) (-9.6%), Haryana (HR) (-3.1%), Uttarakhand (aka Uttaranchal) (UC) (-2.1%), and Maharashtra (MH) (-2.0%) showed a decrease in full vaccination coverage between NFHS-3 and NFHS-4.

**Table 4:** Change in full vaccination between NFHS-3 vs. NFHS-4, and the disparity in full vaccination between > 2 and < 2 risk factors by Indian state.

**State**	**Difference in full vaccination coverage between NFHS-3 vs. NFHS-4**	**NFHS-3:** **Difference in full vaccination between > 2 and < 2 risk factors**	**NFHS-4:** **Difference in full vaccination between > 2 and < 2 risk factors**
***Overall***	*16.2%*	*−13.0%*	*−5.6%*
**AP**	16.0%	−13.0%	0.2%
**AR**	3.8%	−20.0%	−3.8%
**AS**	15.0%	−15.0%	−15.2%
**BH**	32.1%	−24.0%	−3.1%
**DL**	3.2%	11.0%	14.9%
**GJ**	4.8%	−4.0%	−3.9%
**HP**	−9.6%	0.0%	−6.5%
**HR**	−3.1%	0.0%	4.4%
**JH**	26.9%	−16.0%	−12.0%
**JM**	7.1%	−14.0%	5.4%
**KA**	6.8%	−5.0%	−4.6%
**KE**	3.5%	−2.0%	0.0%
**MG**	28.5%	−16.0%	−8.2%
**MH**	−2.0%	−15.0%	−5.4%
**MN**	13.6%	−26.0%	−12.9%
**MP**	10.5%	−19.0%	−8.6%
**MZ**	4.4%	0.0%	7.9%
**NA**	16.0%	−17.0%	−6.2%
**OR**	23.1%	−7.0%	−21.8%
**PJ**	26.3%	−10.0%	3.3%
**RJ**	28.5%	−4.0%	5.1%
**SK**	13.6%	6.0%	−7.0%
**TN**	−14.0%	−4.0%	−5.2%
**TR**	3.6%	−26.0%	−25.2%
**UC**	−2.1%	−16.0%	2.3%
**UP**	29.7%	−8.0%	−2.6%
**WB**	15.4%	−6.0%	−26.5%

## Discussion

Understanding the role of sociodemographic and economic risk factors for childhood non-vaccination is essential to achieving the daunting task of ensuring full vaccination of all infants and children in India, the country with the largest annual birth cohort globally [20]. This study examined the role of different combinations of four key risk factors (infant sex, birth order, maternal education level, and family wealth status) in the context of differences across Indian states among children 12–23 months to assess how equity factors into vaccination rates across states. Nationally, the greater number of risk factors was associated with correspondingly lower rates of vaccination, with this pattern present in 2005–06 and 2015–16. This information can help inform immunization policy by identifying which infants, based on their risk factor profiles, should be prioritized in public health strategies designed to address vaccination inequities among infants.

In order to improve immunization coverage in India, the Government in India introduced a new initiative, Mission Indradhanush in December 2014 with the intent of achieving 90% full immunization coverage across 27 states and union territories by 2020 [21]. The high focus districts identified by the Government of India, which are generally more densely populated and poorer, were largely located in Uttar Pradesh (55 high focus districts) and Bihar (19 high focus districts) and accounted for 38% and 10% of the total completely unvaccinated children, respectively [21]. Another 30% of this total lived in 61 high focus districts located in five other Indian states: Maharashtra, Rajasthan, Gujarat, Madhya Pradesh and Assam [21]. Although phase I (2015) and phase II (2015–2016) of Mission Indradhanush contributed to a 6.7% increase in full immunization coverage, this increase was not sufficient to achieve 90% full immunization coverage among infants [22].

The NFHS-4 data collection period took place during the same time as the initial launch of Mission Indradhanush. Although vaccination coverage in Bihar and Uttar Pradesh increased by 32.12% and 29.74%, respectively, between NFHS-3 (2005–2006) and NFHS-4 (2015–216) data collection periods, these were two of the more populous states in the NFHS-4 that experienced statistically significant disparities in full vaccination coverage, in addition to Madhya Pradesh. Although vaccination coverage improved in the three aforementioned states, disparities in vaccination rates are still apparent among children in India as Mission Indradhanush targeted states largely based on geographic characteristics rather than targeting highest risk children within risk clusters.

The Intensified Mission Indradhanush subsequently commenced in 2017 in follow up to aggressively target 118 districts, 17 urban areas, and 52 districts in North-Eastern India [22]. This program was designed to be monitored at the district, state, and central level with regular benchmarks, in addition to being closely followed under the Pro-Active Governance and Timely Implementation, an information and communication technology platform within the prime minister’s office, at the highest level [22]. There were 24 states targeted* including some of the most populous and poorest like Uttar Pradesh and Bihar [22].

Of the Indian states targeted by the Intensified Mission Indradhanush, Haryana, Maharashtra, and Uttarakhand showed a decrease in full vaccination coverage between NFHS-3 and NFHS-4 whereas Delhi, Gujarat, Jammu & Kashmir, Kerala, Karnataka, Madhya Pradesh, Arunachal Pradesh, Mizoram, and Tripura showed modest increases in full vaccination coverage. Reasons for declining vaccination coverage are likely manifold. We found significant disparities by risk factor count in Maharashtra, and another study found substantial variation within Haryana across districts [23]. These findings speak to the difficulty in equitably distributing vaccines, and that coverage of vaccines statewide may mask substantial differences within the state by geographical region or socioeconomic group.

Otherwise, the Indian states of Odisha, Rajasthan, Jharkhand, Meghalaya, Uttar Pradesh, West Bengal, Assam, Manipur, Nagaland, and Sikkim demonstrated substantial increases in vaccination coverage between the two surveys. Importantly, despite the increases in vaccination coverage observed in some states, they nonetheless continued to experience significant disparities in full vacation coverage among infants. Therefore, the implementation of the Intensified Mission Indradhanush throughout various urban areas and districts in the identified states showed a varied and uneven impact across Indian states as some states showed a moderate to substantial increase in full vaccination coverage, while other states showed a decrease in full vaccination coverage. Results from this study show how vaccination coverage varies substantially geographically across states. Many of the states with low vaccination coverage, including those in central (Madhya Pradesh) and northeast India (Arunachal Pradesh, Assam, Manipur, Nagaland), also have a large proportion of children with 3 or 4 risk factors. Accordingly, although more intensified regional programs to increase vaccination coverage may be warranted, it will be important to maintain equity potentially targeting individual Indian states.

The arrival of the COVID-19 pandemic has also impacted non-pandemic, routine childhood vaccination services [24], and future research can identify if the pandemic has exacerbated disparities. Based on the results of this study, future research on vaccination inequities among infants in India should include a consideration of the negative cumulative impact of multiple risk factors on achieving full vaccination status. It may be strategies to improve childhood vaccination in India should focus on child populations with the greatest probability of experiencing risk clusters at the state or village level. Successful public health programs aimed at improving national vaccination coverage must also address the need to reduce inequities in childhood vaccination rates. Evidence from nearby countries – Bangladesh, Bhutan, Maldives, Nepal, and Sri Lanka – reveals other examples of how routine immunization coverage can be increased. For example, expanding the use of community health workers in administering vaccines, conducting regular assessments of the supply chain, and using local NGOs to generate demand for vaccines and counter vaccine misinformation [26].

### Strengths and limitations

This analysis focused on various combinations of four primary risk factors but did not include religion or caste although they have been shown to be predictors of childhood vaccination [7]. In NFHS-3, Uttar Pradesh, Maharashtra, and Madhya Pradesh accounted for 23.3% of the infants in the survey. In NFHS-4, a total of 3 states – Bihar, Madhya Pradesh, and Uttar Pradesh accounted for 35.4% of infants. Maharashtra and Madhya Pradesh had some of the lowest vaccination coverage in NFHS-3 when examining disparities by full vaccination by number of risk factors**.** These states may have skewed the overall national data rates presented for NFHS-3 and NFHS-4. However, in NFHS-4 there was a significant increase in full vaccination coverage rates in Bihar (32.12%), Madhya Pradesh (10.52%), and Uttar Pradesh (29.74%).

## Conclusions

The results of this study revealed an overall disparity in full vaccination status by the number of risk factors among infants 12–23 months, which was especially evident in the most populous and poorest Indian states like Uttar Pradesh and Bihar. However, these disparities were not as apparent or were absent in many more southern Indian states. Despite governmental efforts to increase infant vaccination coverage through programs like the Intensified Mission Indradhanush, which achieved uneven success, inequities in full vaccination status among infants persist in many Indian states. Although there was an encouraging decrease in vaccination inequities between the two NFHS surveys, some states experienced an increase; thus, new approaches to fully address and hopefully, one day eliminate inequities in full vaccination status among Indian infants are critical.

## Author Contributions

O.K.G contributed to formal analysis and writing of the original draft. A.L.W., J.L.M., and M.L.B. contributed to conceptualization, methodology, and critically reviewed and edited the manuscript for important intellectual content. D.R. contributed to formal analysis and critically reviewed and edited the manuscript for important intellectual content. All authors have agreed to be guarantors of the manuscript as submitted.

## Funding

Research reported in this publication was supported by the National Institute on Minority Health and Health Disparities of the National Institutes of Health under Award Number T37MD001425. The content is solely the responsibility of the authors and does not necessarily represent the official views of the National Institutes of Health.

## Declaration of Competing Interest

The authors declare the following financial interests/personal relationships which may be considered as potential competing interests: Matthew L. Boulton reports financial support was provided by National Institute on Minority Health and Health Disparities of the National Institutes of Health.

## Data Availability

Data will be made available on request.

## Footnotes

* Andhra Pradesh, Arunachal Pradesh, Assam, Bihar, Delhi, Gujarat, Haryana, Jammu & Kashmir, Jharkhand, Karnataka, Kerala, Madhya Pradesh, Maharashtra, Manipur, Meghalaya, Mizoram, Nagaland, Odisha, Rajasthan, Sikkim, Tripura, Uttar Pradesh, Uttarakhand, and West Bengal
